# Bat diversity in the Cuc Phuong National Park, Vietnam - Results from VIETBIO field training and annotated species list.

**DOI:** 10.3897/BDJ.12.e119704

**Published:** 2024-04-29

**Authors:** Sofía I. Hayden Bofill, Frieder Mayer, Vu Dinh Thong

**Affiliations:** 1 Museum für Naturkunde Berlin, Leibniz-Institut für Evolutions- und Biodiversitätsforschung, Invalidenstraße 43, 10115 Berlin, Germany Museum für Naturkunde Berlin, Leibniz-Institut für Evolutions- und Biodiversitätsforschung Invalidenstraße 43, 10115 Berlin Germany; 2 Humboldt-Universität zu Berlin, Lebenswissenschatfliche Fakultät, Unter den Linden 6, 10099 Berlin, Germany Humboldt-Universität zu Berlin, Lebenswissenschatfliche Fakultät Unter den Linden 6, 10099 Berlin Germany; 3 Institute of Ecology and Biological Resources, Vietnam Academy of Science and Technology (VAST), 18 Hoang Quoc Viet Road, Cau Giay district, Hanoi, Vietnam Institute of Ecology and Biological Resources, Vietnam Academy of Science and Technology (VAST) 18 Hoang Quoc Viet Road, Cau Giay district, Hanoi Vietnam; 4 Graduate University of Science and Technology, VAST, 18 Hoang Quoc Viet Road, Cau Giay district, Hanoi, Vietnam Graduate University of Science and Technology, VAST 18 Hoang Quoc Viet Road, Cau Giay district, Hanoi Vietnam

**Keywords:** biodiversity, Chiroptera, genetics, morphology, echolocation

## Abstract

**Background:**

Biodiversity surveys are essential for both academic research and conservation. Integrative approaches that combine morphological, genetic and acoustic aspects for species identification can provide reliable information in taxonomy and evolution. This is especially relevant for those groups with a high degree of cryptic diversity such as bats.

**New information:**

Here, we present the results from a field survey carried out in the Cuc Phuong National Park (CPNP) during 2019 as part of the VIETBIO project and from the examination of specimen collections preserved at the museums of CPNP and the Institute of Ecology and Biological Resources (IEBR). In addition, we include an annotated species list, based on this survey and a literature review. We here confirm that CPNP is home to at least 47 bat species belonging to 23 genera and seven families. We recorded ten of these bat species during our field survey. Obtained data in genetics (sequencing a fragment of the mitochondrial gene COI) supported the morphological identification of the recorded species for which we were able to produce these data. In addition, we include echolocation recordings obtained during our field training with the hope that they may contribute valuable insights to future work concerning the surveyed species. Results from the field survey represent a relevant contribution to biodiversity assessment efforts and, thus, support conservation and management efforts to maintain bat diversity in Vietnam.

## Introduction

### Bat diversity in Vietnam

As a tropical country within Southeast Asia with a long extension from north to south, Vietnam harbours an exceptional high diversity of bats including several endemic species (Huynh et al. 1994, Thong et al. 2012, Kruskop 2013, Thong et al. 2021). With over 1400 bat species recognised worldwide, Vietnam is a country native to approximately 130 bat species (Kruskop 2013, Wilson and Mittermeier 2019, Thong et al. 2021, Thong 2021, Simmons and Cirranello 2023). The variety in climatic conditions throughout Vietnam, as well as its altitudinal heterogeneity, partially led to highly diverse ecosystems for bats. Over the last two decades, scientific and conservation efforts have focused on characterising and delineating the bat diversity in Vietnam (Hendrichsen et al. 2001, Borisenko and Kruskop 2003, Borisenko et al. 2009, Can et al. 2008, Kruskop 2013, Thong et al. 2021) and, although these efforts have greatly contributed to our knowledge of Vietnam’s bat biodiversity, the taxonomy, distribution and conservation status of many species are still unclear due to the complex and diverse topography and ecosystems. In addition, assessment of bat diversity is a serious challenge since many bat taxa are morphologically cryptic or might encompass more than one phylogenetic lineage (Borisenko and Kruskop 2003, Kruskop 2013, Thong et al. 2021, Yuzefovich et al. 2022). Bats play crucial ecological roles in the maintenance of environmental stability as pollinators, seed dispersers and controllers of insects (Kunz et al. 2011, Ramírez-Fráncel et al. 2022). Moreover, the guano produced by bats, used as fertiliser, highlights their economic importance (Sakoui et al. 2020). Biodiversity decline, likely driven by factors such as habitat fragmentation and climate change, contributes to the endangerment of bats (Festa et al. 2022, Tuan et al. 2023). Therefore, bat diversity assessments are fundamental to provide information for conservation and management efforts and to understand how habitat fragmentation and climate change affect bat diversity in a country with such an immense species richness.

### Cuc Phuong National Park

Cuc Phuong National Park (CPNP) is located in north-eastern Vietnam, approximately 120 km southwest from Hanoi. With a total area of 22,200 ha, the Park covers three provinces: Ninh Binh, Hoa Binh and Thanh Hoa (Cuc Phuong National Park 2011). Prior to 2019, at least 59 bat species inhabiting the Park were included in previous publications and grey literature (Howard and Hill 1999, Khoi et al. 2001, Feiler et al. 2008). However, the taxonomic status of many species included in previous publications has been changed following recent studies incorporating genetics (Yuzefovich et al. 2021, Yuzefovich et al. 2022). On the other hand, many previous records from Cuc Phuong National Park were obtained from provisional identification of specimens collected over general faunal surveys (Howard and Hill 1999, Cuc Phuong National Park 2011). To our knowledge, the latest species composition of bats from Cuc Phuong National Park was published by Khoi et al. (2001). Several subsequent publications just included records of referential materials in a target research or review (Hendrichsen et al. 2001, Borisenko and Kruskop 2003, Thong et al. 2012a, Thong et al. 2012b, Kruskop 2013, Thong et al. 2021, Thong 2023). As mentioned above, the change of taxonomic status of different species, which were recorded from CPNP, indicated the need for a revision of bats from Cuc Phuong to confirm the bat diversity within the National Park following the current systematics and nomenclature procedures.

In this data paper, we provide the results from our 2019 field survey and an annotated list of species present in CPNP, based on three information sources: literature review, identification of bats captured over the survey to CPNP in the context of the VIETBIO project (Duwe et al. 2022) and examination of specimens preserved at the Park’s museum. Acoustic recordings of ten surveyed echolocating bat species are also included in this paper (see Additional Information).

## Sampling methods

### Study extent

During our survey at the CPNP in May 2019 (see Temporal Coverage below for exact dates), we sampled 32 bat individuals belonging to ten species of five genera, and three families at seven different collecting sites/events with the purpose of confirming the presence of bat species previously recorded in the Park, as well as providing new or supplemental data of as many different bat species as possible. The study sites comprise representative ecosystems of the Park including streams, cave, primary forests on karst and secondary forests located in an elevation range from 35 to 275 m above sea level. From the 32 sampled individuals, 30 were identified by their morphology up to species level and two up to only genus level (see Suppl. material 3).

### Sampling description

**Bat capture**

**Figure 1.**
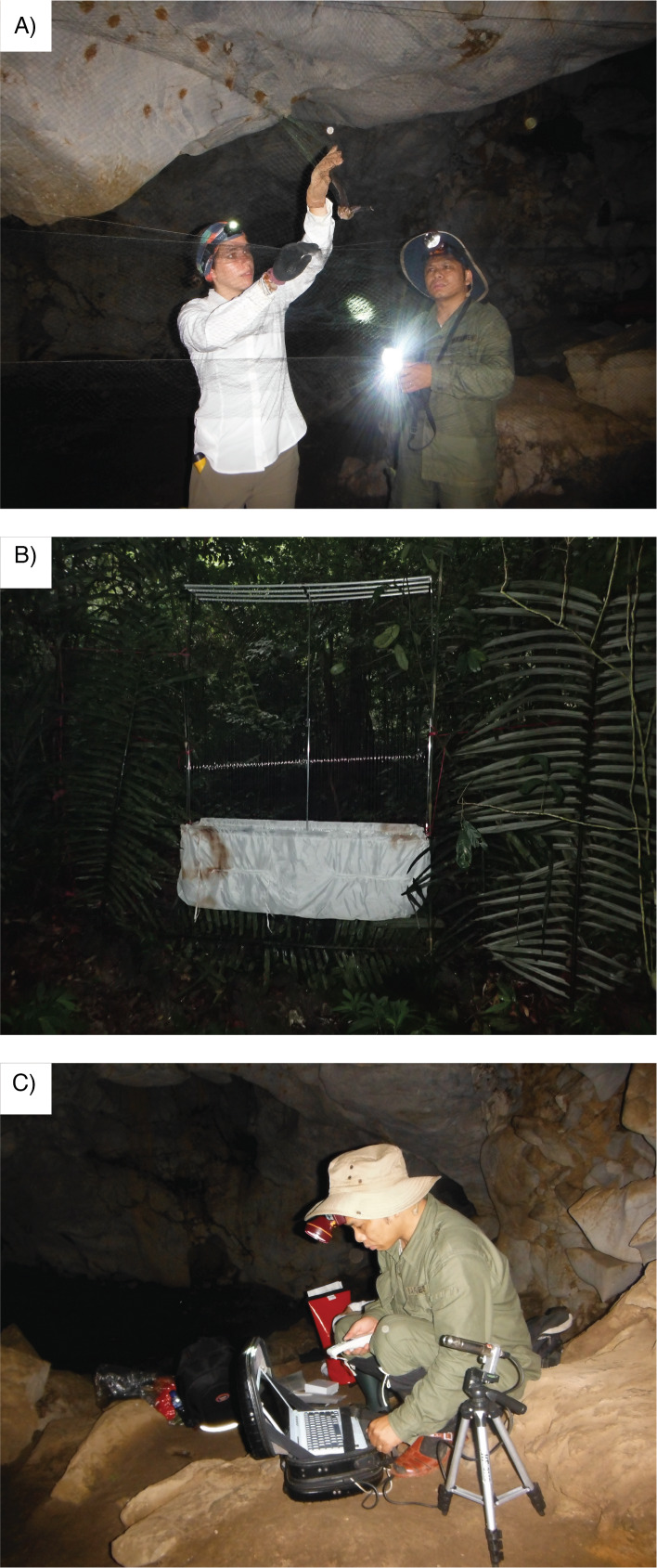
Methods used for capturing bats and recording echolocation calls during our field survey. In a) mist-netting bats in a cave; b) harp-trap setup in the forest and c) recording of echolocation calls in a cave. Photographs by Vu Dinh Thong and Luong Khac Hien.

Bats were captured and handled following the guidelines recommended by the American Society of Mammalogists (Kurta and Kunz 1988, Thomas and West 1989, Jones et al. 1996, Sikes et al. 2011, Sikes and the Animal Care and Use Committee of the American Society of Mammalogists 2016). Bats were captured using mist nets (Ecotone, Poland) and a harp trap (Fig. 1a and b). Each mist net consisted of a fine nylon mesh separated into 3 - 5 shelves, which formed pockets when the bat flew against them, ensuring entanglement of each netted bat. The net sizes (3-5 m height x 8-20 m length) were used according to the space and habitats of the netting sites. Bamboo or inox poles (ca. 3-6 m length) were used to set the net, while nylon ropes were used to stabilie and secure the net on a nearby tree or to the ground with the help of securing stakes. Each harp trap consists of four dismountable metal frames (2.0 m [height] x 1.5 m [width]) separated from each other by 15 cm (Francis 1989). Each frame had vertical lines of thin wires of monofilament fishing lines, fastened 2.5 cm apart. After bats flew against the harp trap, they fell into a bag, which had internal plastic flaps to prevent bats from flying or crawling out. Mist nets were used primarily inside caves (8 m and 12 m in length), as well as across trails (8 m in length) and bodies of water (20 m in length). In contrast, harp traps were utilised along narrow pathways and streams located within densely-vegetated patches of forest where the setup of mist nets posed significant challenges.

Each captured bat individual was kept in a cotton bag and weighed using a spring scale. Forearm length was measured using a Mitutoyo 0-200 mm Digital caliper with 0.01 mm digital steps. Reproductive status and age were assessed following Racey (2009) and Brunet-Rossini and Wilkinson (2009), respectively. Two wing punches, taken from right and left wing, were collected from each representative individual of the captured taxa using a 3 mm or a 5 mm wing puncher and samples were preserved in 90% pure ethanol in labelled 1.5 ml tubes. Photographs of each individual were taken using a digital camera (Cannon 60D). Bats were processed immediately during the night of capture and were released at capture site after taking the selected measurements. Echolocation calls were recorded while bats were released. Captures and processing started around 5:30 pm and lasted until approximately 12 am of the same night or 1 am of the subsequent day. In addition, wing punches and morphological measurements from museum specimens conserved in fluid (formalin) at the Museum of the Cuc Phuong National Park were taken.

**Echolocation recording**

Sound recordings were carried out in the field at three different situations: (1) bats were flying in their natural habitats at night, (2) flying inside a flight tent (4 m [length] x 4 m [width] x 3 m [height]) and (3) during release at capture site. Echolocation calls were recorded using a PCTape system with a sampling rate of 480 kHz. Batman software was used to obtain high-quality sound sequences since it displays colour sonograms of the detected echolocation signals in real-time. Detected echolocation signals were displayed as sonograms in real time with Selena software using a 512-size Fast Fourier Transformation. The PCTape system, Batman and Selena software are custom-made by the University of Tübingen, Germany. An SM4BAT FS recorder produced by the Wildlife Acoustics (USA) with sampling rate up to 500 kHz was also used to record echolocation calls of bats in natural habitats (Fig. 1c). While no processing of echolocation calls is presented in this data article, echolocation recordings are included in the dataset to facilitate accessibility for future, more extensive studies focusing on bat acoustics and diversity within CPNP (for example, see Thong (2023)).

**Molecular data processing**

In order to genetically corroborate the species taxonomy and support the morphological identifications in the field, we generated gene sequences from the collected samples at the Museum für Naturkunde Berlin - Leibniz Institute for Evolution and Biodiversity Science (MfN). DNA was isolated from wing punches preserved in ethanol with a salt/chloroform procedure (Dietz et al. 2016) or with the Qiagen DNA Isolation DNeasy Blood and Tissue Kit (Qiagen©). Samples were lysed in a thermomixer for one hour at 56°C with a lysis buffer and proteinase K and left overnight at 37°C. DNA isolation was continued on the following day. DNA quality checks were carried out in an electrophoresis chamber after each isolation on a 1% agarose gel at 120V. After successful isolation, the mitochondrial gene COI (657 bp) was amplified using a polymerase chain reaction (PCR) with the primers VF1d (5'-TTCTCAACCAACCACAARGAYATYGG-3') and VR1d (5'-TAGACTTCTGGGTGGCCRAARAAYCA-3') (Ivanova et al. 2007, Lim et al. 2016). PCRs were conducted in 25 μl reaction volumes containing 16.9 μl of water, 2 μl DNA, 1 μl of each primer (10 μM), 2.5 μl Buffer (10x), 0.5 μl dNTPs (10 mM), 1 μl MgCl_2_ (25 μM) and 0.1 μl Taq DNA Polymerase (5000 U/ml)). PCRs were performed in a thermocycler. The PCR-programme started with an initial denaturation at 94°C for 3 minutes. After this initial step, a cycle composed of the following steps initiated: 1) a short denaturation at 94°C for 30 seconds; 2) annealing of the primers to the DNA at 45°C for 40 seconds; 3) an extension step at 72°C for 1 minute and 30 seconds. This cycle was repeated 40 times. The final extension step was carried out at 72°C for 10 minutes. PCR products were stored at 4°C. Thirty DNA sequences were generated by Macrogen (Macrogen Europe, Amsterdam, the Netherlands).

Editing and alignment of sequences was done with Geneious Prime (v.2019.2.3) (http://www.geneious.com). We visually inspected and edited the gene sequences, for which ends were trimmed and low quality sections corrected. After edition, sequences were blasted and compared with the GenBank (Benson et al. 2012) and Barcode of Life Data System (BOLD Systems) (http://www.barcodinglife.org, Ratnasingham and Hebert 2007) databases. We visualised the generated gene sequences to published references by first aligning the sequences with Muscle 5.1 (Edgar 2004) and then reconstructing a Neighbour-joining tree, based on the Tamura-Nei genetic distance model with 100 replicates. Sequences are published in GenBank under the accession numbers OR950725 - OR950751.

## Geographic coverage

### Description

Vietnam: Cuc Phuong National Park.

### Coordinates

20.2441 and 20.3939 Latitude; 105.7299 and 105.5200 Longitude.

## Taxonomic coverage

### Description

Thirty-two bat individuals belonging to ten species, five genera and three families were sampled in seven field sites (Suppl. material 3), of which, 30 individuals were identified to species level in the field, based on their morphological characteristics and measurements. Two small horseshoe bat individuals were identified as "Rhinolophuscf.pusillus” in the field because of unclear diagnoses in morphology of every member belonging to this species group. Succesfully generated COI sequences for one of those two small horseshoe bats enabled the confirmation of the surveyed individual as *Rhinolophuspusillus*. We generated 28 COI sequences and compared those with reference sequences from previous studies. The produced COI sequences are published in GenBank and include amongst them a sequence for *Hipposiderosalongensissungi*, a globally endangered subspecies, endemic to Vietnam.

Amongst the surveyed species, *Hipposiderospoutensis* was the most commonly captured species (ten individuals surveyed), followed by *Hipposiderosgentilis* (seven individuals captured). *Hipposiderosalongensissungi* and *Aselliscusstoliczkanus* were captured only two and four times, respectively. The remaining species were rarely captured and recorded over the surveys: *Rhinolophusaffinis*, *Rhinolophuspusillus*, *Rhinolophusthomasi*, *Rhinolophussiamensis*, Murinacf.cyclotis and *Iaio*, with sightings ranging from one to two individuals (see Suppl. material 1).

**Taxonomic comments**

Hipposideridae

**Figure 2.**
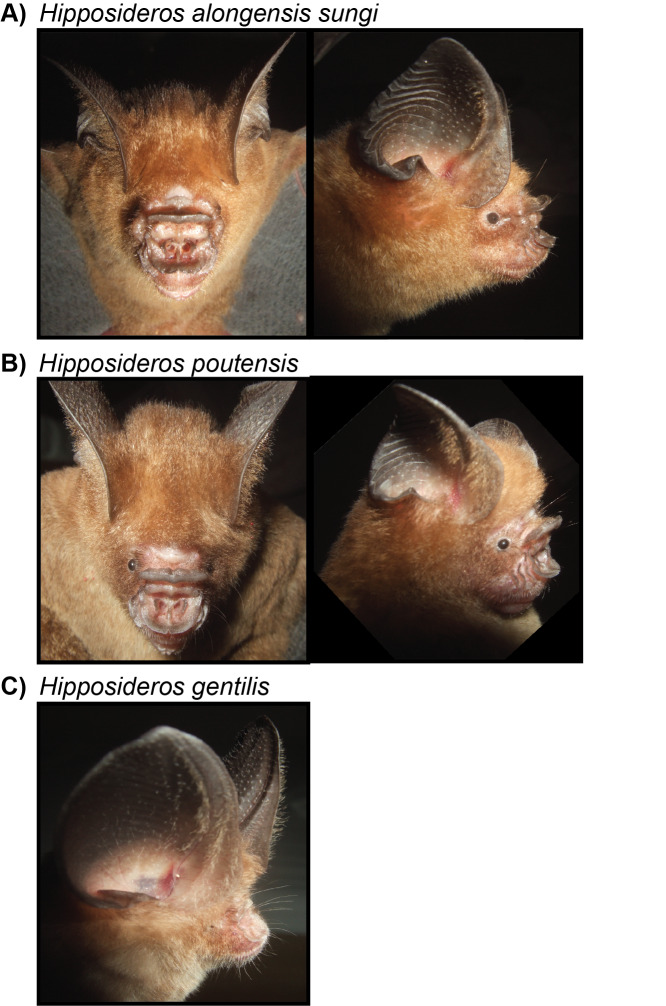
Photographs of three of the four surveyed hipposiderid species: a) *Hipposiderosalongensissungi*; b) *Hipposiderospoutensis* and c) *Hipposiderosgentilis*. Photographs by Vu Dinh Thong.

Four species belonging to this family were captured: *Aselliscusstoliczkanus*, *Hipposiderosalongensissungi*, *Hipposiderosgentilis* and *Hipposiderospoutensis* (Fig. 2). Morphological characteristics of the captured individuals exhibited well the diagnoses of this taxon, allowing us to clearly distinguish each taxon from the other. For example, the three sampled *Hipposideros* species have distinct, non-overlapping body sizes and forearm lengths, with *H.gentilis* (38 - 43 mm forearm length) being the smallest amongst the three, *H.poutensis* the intermediate form (50 - 67 mm forearm length) and *H.alongensissungi* being the largest species (68 - 76 mm forearm length) (Wilson and Mittermeier 2019,Yuzefovich et al. 2022) . The species *A.stoliczkanus*, although similar in forearm length to *H.gentilis* (39 - 45 mm), is distinguished by the presence of a tridentate noseleaf, facilitating its differentiation from the rest of the surveyed hipposiderid bats. These species inhabit similar habitats and were captured at caves and other capture sites under forest canopy. The taxonomic status of the four taxa (*A.stoliczkanus*, *H.poutensis, H.a.sungi* and *H.gentilis*) was genetically confirmed, based on publicly available sequences in GenBank and BOLD. The available COI sequences in the databases for *H.alongesissungi, H.poutensis* and *H.gentilis* still follow earlier taxonomic opinion (identify as *Hipposiderosturpis alogensis, Hipposideroslarvatus* and *Hipposiderospomona*, respectively), but high sequence similarity allowed the confirmation of the identifications. The four species have been recorded in the Park over the past decades, but *H.poutensis* and *H.gentilis* were identified as *H.larvatus* and *H.pomona*, respectively (Thong 2023).

Rhinolophidae

**Figure 3.**
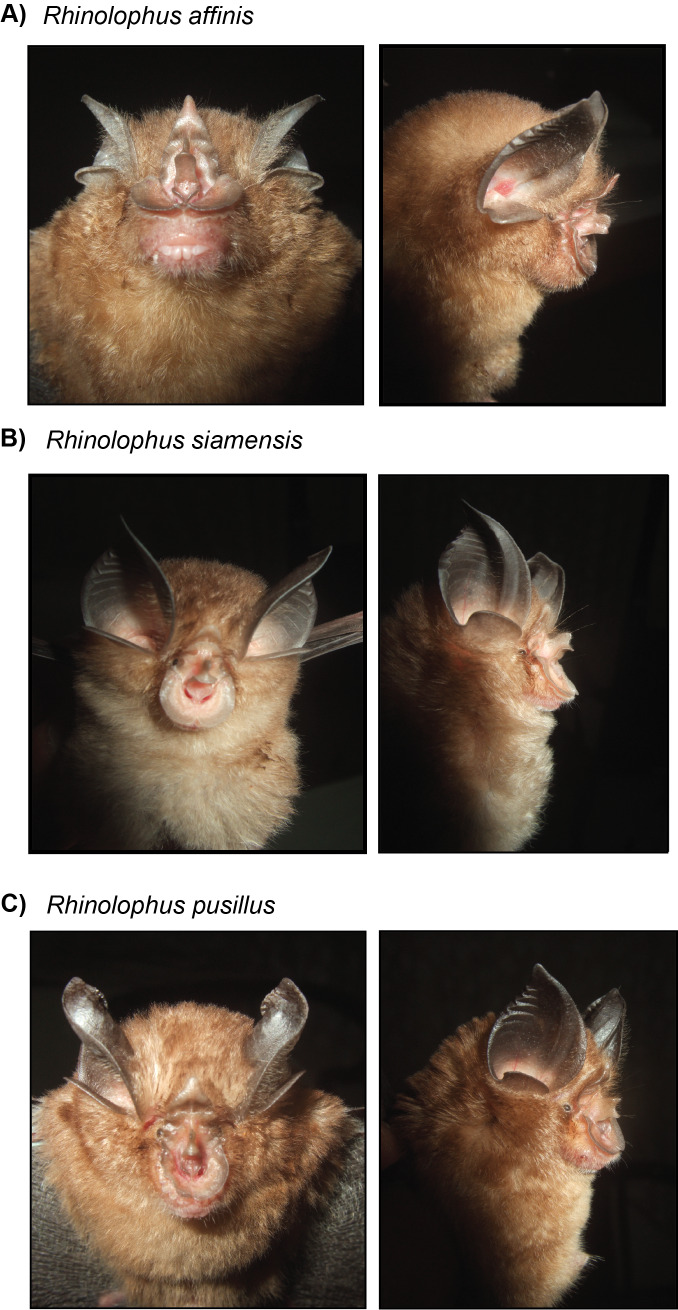
Photographs of three of the four sampled horseshoe bat species. In a) *Rhinolophusaffinis*; b) *Rhinolophussiamensis* and c) *Rhinolophuspusillus*. Photographs by Vu Dinh Thong.

We collected four species from this family: *Rhinolophusaffinis*, *Rhinolophuspusillus*, *Rhinolophusthomasi* and *Rhinolophussiamensis* (Fig. 3). These four species were captured under forest canopy. The taxonomic status of one of the two small horshoe bats was confirmed as *R.pusillus* with reference to both morphological and genetic data (99.6% Pairwise Identity). We were unable to generate a COI sequence for the second individual, but their morphological traits were highly similar. Moreover, the morphological characteristics of a firstly unidentified horseshoe bat individual are almost identical to those of *R.siamensis*; however, its mitochondrial COI barcode (GenBank Accession number OR950749 , see Suppl. material 3) obtained equal support for the identification of the sequence as *R.huananus*, *R.siamensis* (99.8% Pairwise Identity in NCBI) and *R.macrotis* (99.7% Pairwise Identity). Following recent phylogenetic studies, the taxonomy of the *macrotis* species group has been subject to relevant changes (Tu et al. 2017, Zhang et al. 2018, Liu et al. 2019, Wilson and Mittermeier 2019). Currently, the macrotis species group comprises seven species (*R.macrotis*, *R.siamensis*, *R. osgoodi, R. episcopus, R. marshalli, R. schnitzleri and R. rex*), with distinguishable biogeographic, genetic and morphological characteristics (Liu et al. 2019, Wilson and Mittermeier 2019). In light of these findings and taking into consideration that *R.huananus* represents a junior synonym of *R.siamensis*, the *Rhinolophus* individual was confirmed genetically as *R.siamensis*. Moreover, the *R.affinis* individual could primarily be identified by its morphology, as we were not able to successfully generate a complete COI sequence appropiate for submission (sequence 343 bp long, blast 99% Pairwise Identity match to *R.affinis* reference sequences). Additional sampling efforts for both morphometric and genetic data of the species from CPNP are required for taxonomic confirmation in the coming time.

Vespertilionidae

**Figure 4.**
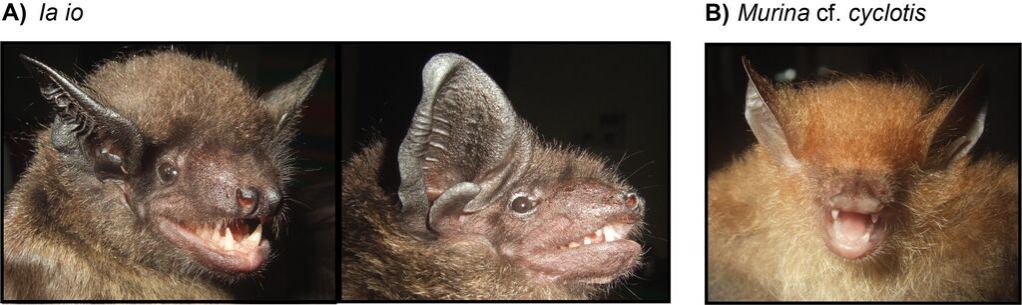
Photographs of two sampled vesper bat species: a) *Iaio* and b) Murinacf.cyclotis. Photographs by Vu Dinh Thong.

Two individuals of this family were captured over our survey in 2019: one is identified as Murinacf.cyclotis and the second is identified as *Iaio* (Fig. 4). The Murinacf.cyclotis individual was found in a dense patch of forest. To date, *Murinacyclotis* is the only species of *Murina* recorded from CPNP and, although no genetic sequence was successfully produced for this individual, morphological characteristics are almost identical to descriptions of *Murinacyclotis* in previous publications. For this reason, the captured individual is provisionally identified as Murinacf.cyclotis. The individual identified as *Iaio*, one of the largest Asian vesper bat species and classified as near threatened, was found in a deep and large cave. This is the only species in the genus *Ia* and has been previously reported to occur in CPNP.

**Discussion**

**Figure 5.**
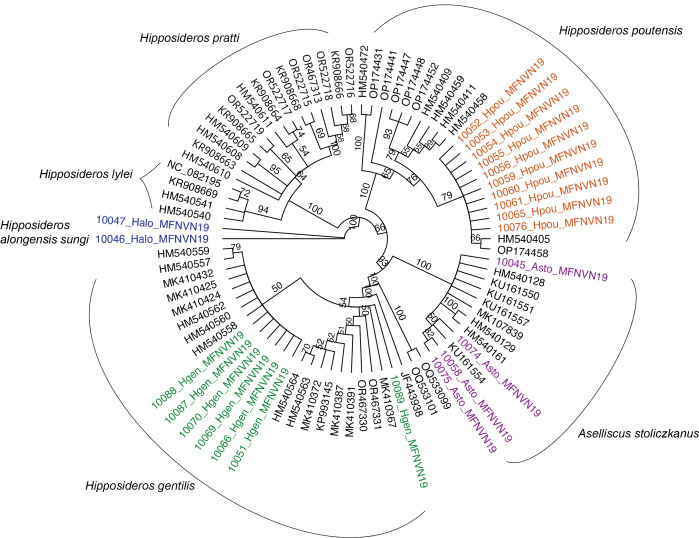
Unrooted Neighbour-joining tree of the Hipposideridae species surveyed during the field training (in colour) and the reference sequences with the highest match found in the databases.

**Figure 6.**
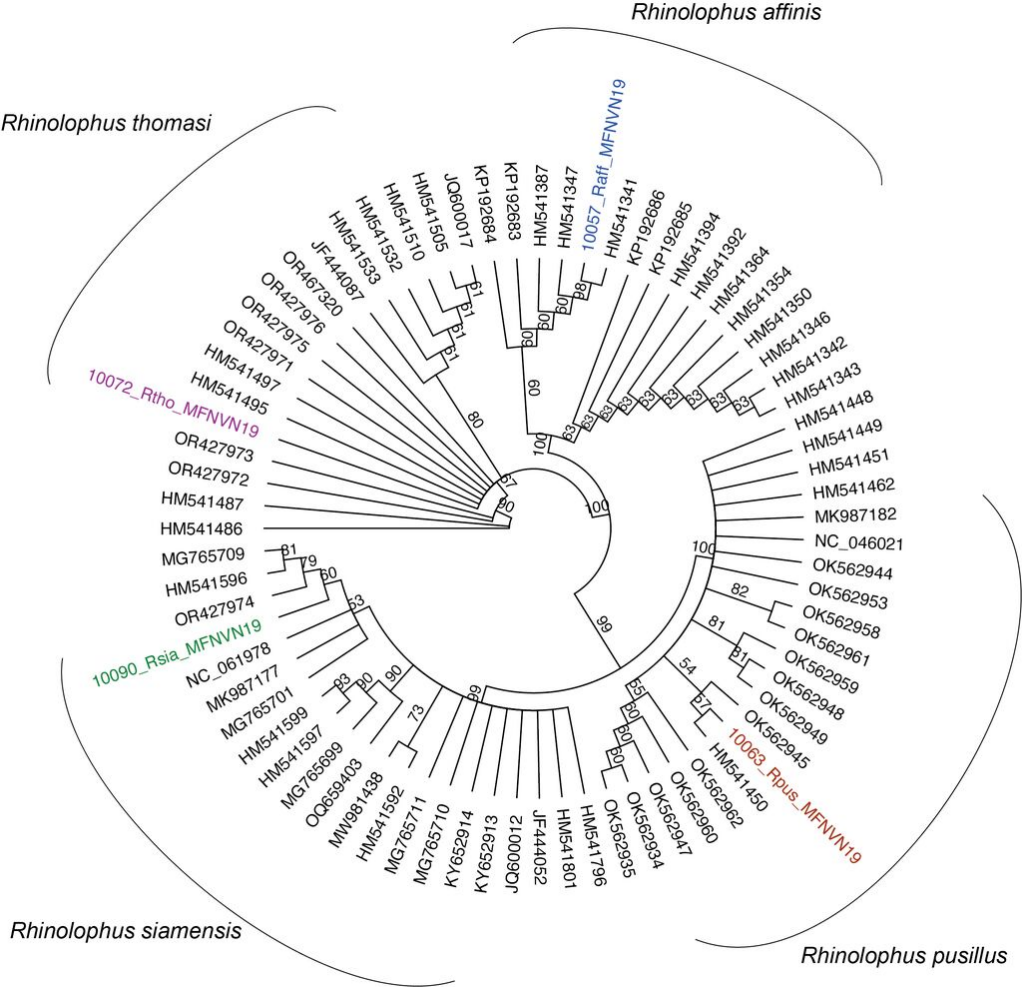
Unrooted Neighbour-joining tree constructed using the generated sequences of the sampled horse shoe bats (in colour) and the references with highest match found in the databases. Partial sequence of the *Rhinolophusaffinis* individual was not sufficient for GenBank submission (343 bp long), but allowed its placement amongst other publicly available *R.affinis* sequences.

**Figure 7.**
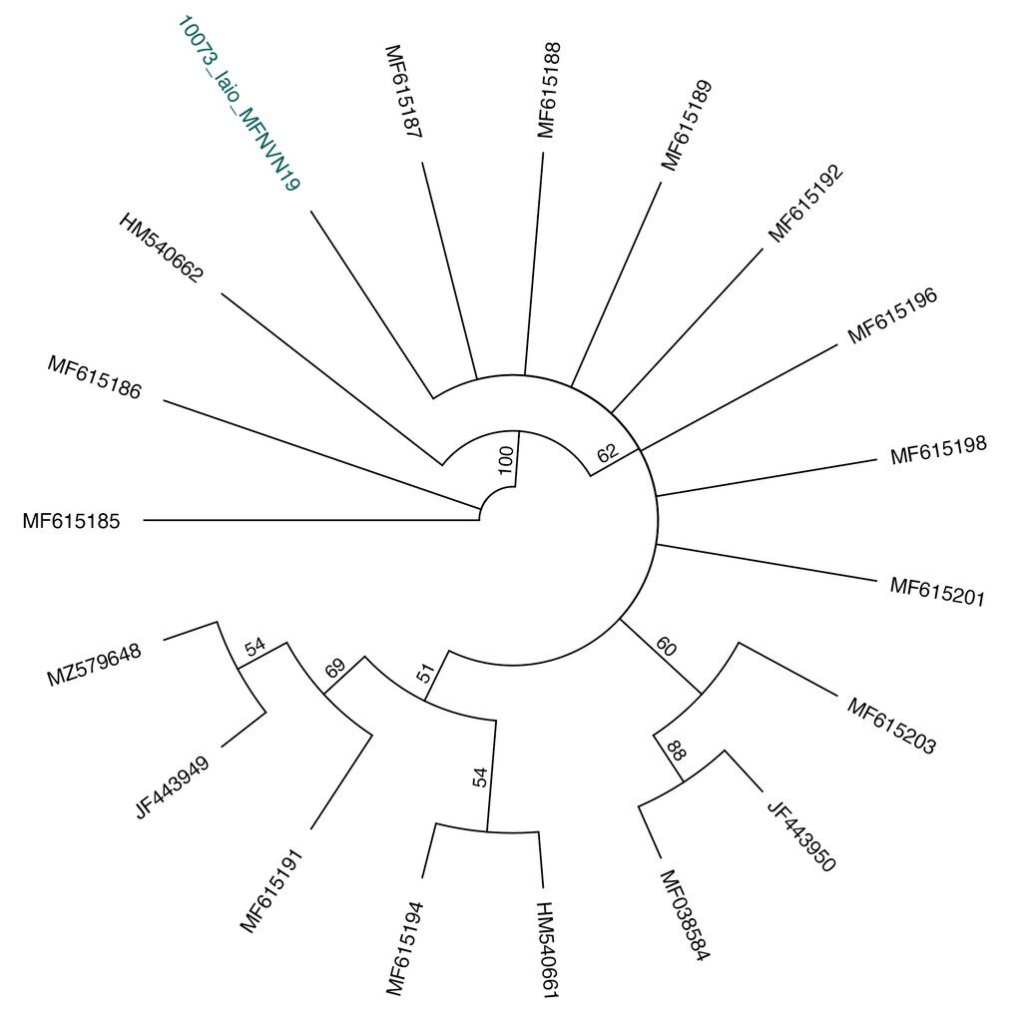
Unrooted Neighbour-joining tree using the generated *Iaio* sequence (in colour) and publicly available sequences for the species.

During our field survey at CPNP in 2019, ten species were captured and recorded at seven netting/trapping sites. Most of the sampled species have widespread distributions, some extending throughout Asia, whereas only the subspecies *H.alongensissungi* is endemic to north-eastern Vietnam. Assessing the occurrence of species in protected areas such as CPNP can provide valuable insights into the relevance of national parks and their role in maintaining biodiversity. The ten identified species have been previously reported to occur in the Park and were identified primarily on their morphology (Huynh et al. 1994, Thomas et al. 1997, Hendrichsen et al. 2001, Khoi et al. 2001, Thong et al. 2012a, Kruskop 2013, Thong 2013, Thong 2023). Nonetheless, this is one of the few documents containing data in morphology and genetics. Morphological identification of nine out of the ten species was validated through sequencing of the mitochondrial COI gene by comparing the barcodes with publicly available gene sequences (Figs 5, 6, 7). We hope these data can contribute to ongoing and future efforts for understanding lineage diversity in Southeast-Asian bat species.

Furthermore, we acknowledge the inherent limitations associated with the methods used for netting/trapping in drawing conclusions regarding species occurrence and abundance. For example, nets and traps enable sampling only small areas in comparison to the broader ranges occupied by free-flying bats and these devices can be detected and circumvented by many bat species (O'Farrell and Gannon 1999, Flaquer et al. 2007). Moreover, bat activity can greatly fluctuacte from one night to another. Increasing the number of sampling sites, capture events (nights) and the diversity of surveyed habitats can contribute to improving the accuracy of bat diversity assessments. In addition, adopting an integrative approach that incorporates sequencing genetic material, such as traditional barcoding or environmental DNA (eDNA) approaches, alongside the recording of echolocation calls, can yield valuable insights into bat diversity and ecology.

Apart from the survey results, we also reviewed available literature containing records or contents relevant to bats of CPNP following the current systematics for confirmation of the Park’s bat diversity (refer to Suppl. material 2). Here, we also include the annotation of the individuals found at the CPNP Museum collection. We found that CPNP is home to at least 47 bat species belonging to 23 genera and seven families. However, recent studies in phylogenetics and taxonomic revisions have resulted in relevant taxonomic modifications within various species groups. Consequently, we acknowledge that there may be uncertainties concerning the completeness and up-to-date nature of some of the material utilised to annotate the bat diversity of the Park. For instance, the CPNP website indicates the presence of *Miniopterusaustralis*; however, current published literature does not support the presence of this species in Vietnam (Kruskop 2013, Thong 2021). We emphasise the need for confirmation of occurrence for many of these species in CPNP.

Although the number of species recorded during our field survey is only a small part of the known species occurring in the Park (Cuc Phuong National Park 2011), we here highlight the relevance in assuming an integrative approach to bat taxonomy and evolution in Southeast Asia. We hope that the data gathered during our survey will contribute valuable taxonomic and occurrence information to future bat biodiversity assessments in Vietnam and the Southeast-Asian Region.

### Taxa included

**Table:** 

Rank	Scientific Name	Common Name
family	Hipposideridae	Old World Leaf-nosed bats
species	*Aselliscusstoliczkanus*	Stoliczka's Trident bat
species	*Hipposiderosgentilis*	Andersen's Roundleaf bat
species	*Hipposiderospoutensis*	Allen's Roundleaf bat
species	*Hipposiderosalongensissungi*	Sung's Leaf-nosed bat
family	Rhinolophidae	Horseshoe bats
species	*Rhinolophuspusillus*	Least Horseshoe bat
species	*Rhinolophussiamensis*	Thai Horseshoe bat
species	*Rhinolophusaffinis*	Intermediate Horseshoe bat
species	*Rhinolophusthomasi*	Thomas's Horseshoe bat
family	Vespertilionidae	Vesper bats
species	Murinacf.cyclotis	Round-eared Tube-nosed bat
species	*IaIo*	Great evening bat

## Temporal coverage

**Data range:** 2019-4-29 – 2019-5-10.

## Usage licence

### Usage licence

Creative Commons Public Domain Waiver (CC-Zero)

## Data resources

### Data package title

Collection Datasheet

### Number of data sets

1

### Data set 1.

#### Data set name

Collection Datasheet

#### Download URL

https://osf.io/dav84/?view_only=7d61203bfb704bf0a7ba0dfe7a14e825

#### Description

File including sampling data, echolocation metadata and genetic sequence information. Echolocation recordings can be found in the zipped folder "CucPhuongAprMay2019.tar.gz" and genetic sequences in NCBI under the accession numbers: OR950725 - OR950751.

**Data set 1.** 

Column label	Column description
Individual field code	Individual record-code-ID from the collector.
Record_type	The type of record.
Collection object type	The type of collection object.
Sex	Sex of specimen.
Life stage	Life stage of specimen.
Specimen_preservation	Preservation of the specimens.
Specimen_preparation	Preparation of the specimens.
Specimen_parts	Type of tissue collected.
Specimen_comments	Comments of specimen.
Deposit_specimen_institution	The actual or presumed institutional depository in case of a specimen-based record.
Deposit_institution_collection	Specimen ID.
Recorder/Collector	The person who collected/observed this specimen. Order of names: surname, first name. In case of several collectors: separated by underscore (NOT semicolon).
Event_date_start (collection date start)	Date when event started (DD-MM-YYYY).
Event_date_end (collection date end)	Date when event ended (DD-MM-YYYY).
Event_year_start	Year when event started (YYYY).
Event_month_start	Month when event started.
Event_year_end	Year when event ended (YYYY).
Event_month_end	Month when event ended.
Recording_method	Method used for collecting data.
Event_record_comments (Collection notes)	Collection notes.
Kingdom	Name of taxon.
Phylum	Name of taxon.
Class	Name of taxon.
Order	Name of taxon.
Family	Name of taxon.
Genus	Name of taxon.
Qualifier	A term to qualify the identification.
Species (Specific Epithet)	Name of taxon; In case of an unresolved species pair or group, enter the species names in alphabetic order, separated with a slash.
Determination_according_to/ Identifier	The person who identified this specimen. Order of names: surname, first name. In case of several collectors: separated by underscore (NOT semicolon).
Determination_date/ Identification Date	Date of identification (DD-MM-YYYY).
Country	The English name of the country of the locality (Standardised spelling for names).
1stLevel_Province_State	The English name of the province of the locality (Standardised spelling for names).
4thLevel_Community_Village	The English name of the community/village of the locality (Standardised spelling for names).
Locality_verbatim	Sampling location.
Locality_description	The full describtion of the locality, indicating important nearby topographic landmarks.
Latitude_decimal	Display in degrees(°) and decimal degrees (hddd.ddddd°).
Longitude_decimal	Display in degrees(°) and decimal degrees (hddd.ddddd°).
Georeference_uncertainty_metres	Uncertainty in metres.
Elevation_minimum_metres	Elevations in metres (m) [m a.s.l.]; If there is no elevation span (minimim and maximum), then enter the elevation here.
Media_type	Type of Media file.
Amount	Amount of files.
Media_file_name	Name of file.
Media_file_2	Type of media 2.
Amount	Amount of type of media 2.
Media_file_name_2	Name of file 2.
Media_created_by	Order of names: surname, first name. In case of several creators: separated by underscore (NOT semicolon).
Seq_marker	Type of sequence marker.
Seq_name	Name of sequence file.
Seq_processed_by	Name of the person who processed the genetic sample.
Seq_generated_by	Institution at which sequence was generated.
Seq_length	Length of sequence after trimming.
Identity_database	Identity percentage in NCBI.
Species_database	Name of species from NCBI database with the highest identity score.
Forearm_length	Bat forearm length in mm.
Tibia	Length of tibia in mm.
Foot_height	Foot height in mm.

## Supplementary Material

D11C81BF-B200-5BB5-ABBD-607C1BFADE2E 10.3897/BDJ.12.e119704.suppl1 Supplementary material 1 Summary Checklist Data type Occurrence File: oo_1032369.xlsx https://binary.pensoft.net/file/1032369 Hayden Bofill, S. I., Mayer, F., and Thong, V. D

A2955A92-2926-5CB2-9483-3FBDF63EC5E4 10.3897/BDJ.12.e119704.suppl2 Supplementary material 2 Annotated list of bat species from Cuc Phuong National Park Data type Annotated species list from Cuc Phuong National Park File: oo_1032365.xlsx https://binary.pensoft.net/file/1032365 Hayden Bofill, S.I., Mayer, F., and Thong, V. D.

F0885D95-3A2A-5992-BFBB-F4F5A3963AC0 10.3897/BDJ.12.e119704.suppl3 Supplementary material 3 Collection Datasheet Data type Collection and sampling details, geographic locations, genetic and acoustic metadata File: oo_1032692.xlsx https://binary.pensoft.net/file/1032692 Hayden Bofill, S. I., Mayer, F., and Thong, V. D.
